# Level of Ascorbic Acid, Total Phenolic, Flavonoid, Tannin, and Antioxidant Activity of Lady Finger (*Abelmoschus esculentus*) From Addis Ababa, Ethiopia

**DOI:** 10.1155/ianc/6835094

**Published:** 2026-09-11

**Authors:** Gezehagn Tola, Weldegebriel Yohannes, Bhagwan Singh Chandravanshi

**Affiliations:** ^1^ Department of Chemistry, College of Natural and Computational Sciences, Addis Ababa University, P. O. Box: 1176, Addis Ababa, Ethiopia, aau.edu.et

**Keywords:** *Abelmoschus esculentus*, ABTS, antioxidant activity, ascorbic acid, DPPH, flavonoids, FRAP, tannin content, total phenolic content

## Abstract

Oxidative stress, resulting from an imbalance between reactive oxygen species and cellular defense mechanisms, is the fundamental driver of various chronic metabolic disorders. Consequently, the consumption of antioxidant‐rich vegetables is essential for public health as a strategy to mitigate free radical–induced damage. This study aimed to evaluate the phytochemical composition and antioxidant potential of lady finger (*Abelmoschus esculentus*) varieties sourced from four representative subcities of Addis Ababa (Arada, Lemi Kura, Bole, and Lafto), Ethiopia. The levels of ascorbic acid were determined by iodometric titration, while total phenolic content (TPC), flavonoid content (TFC), and tannins were quantified using colorimetric methods. Antioxidant capacity was evaluated through three complementary assays: 2,2‐diphenyl‐1‐picrylhydrazyl (DPPH), 2,2′‐azino‐bis(3‐ethylbenzothiazoline‐6‐sulfonic acid) (ABTS), and ferric reducing antioxidant power (FRAP). Ascorbic acid concentrations ranged from 5.27 to 5.88 mg/100 g. The Arada sample exhibited the highest TPC (130.21 mg GAE/100 g) and TFC (243.92 mg QE/100 g), while the Lafto sample had the highest tannin concentration (70.51 mg/100 g). Radical scavenging potency was high across all samples, with IC_50_ values for DPPH ranging from 84.4 to 86.7 μg/mL and ABTS ranging from 63.9 to 92.5 μg/mL. The Bole sample demonstrated the highest overall antioxidant activity, with a FRAP value of 201.4 mmol Fe^2+^/100 g of extract. These findings highlight lady finger as a valuable food source. The high antioxidant capacity suggests that these varieties are rich in dietary antioxidants, potentially enhancing the overall nutritional quality of the diet.

## 1. Introduction

Access to nutrient‐dense food is a critical public health priority, as nutritional quality often lags behind industrial and agricultural production. Vegetables are essential components of a balanced diet, providing vital micronutrients and bioactive phytochemicals that may help prevent chronic diseases [1–11]. Among these, lady finger (*Abelmoschus esculentus*), known locally in Ethiopia as “Bamia,” is highly valued for its adaptability and rich nutritional profile [12–18]. It represents a significant component of global diets not only due to its nutritional value but also for its versatile applications in drug delivery systems, food technology, and healthcare [8]. These health‐promoting benefits are largely attributed to secondary metabolites, such as phenolic compounds and flavonoids, which are reported to exhibit potent antioxidant activities and anti‐inflammatory activities [19–22].

From an epidemiological perspective, low dietary intake of these antioxidants is increasingly linked to the rising global burden of noncommunicable diseases. Nutritional deficiencies in bioactive compounds exacerbate oxidative stress, a condition characterized by an imbalance between reactive oxygen species and the body’s antioxidant defenses. This imbalance leads to systemic cellular damage and is a fundamental driver in the pathogenesis of chronic illnesses, including cardiovascular disease, Type 2 diabetes, and certain cancers [23–26]. Consequently, ensuring adequate intake of antioxidant‐rich vegetables is a key strategy in mitigating the global morbidity associated with these conditions.

Despite the benefits of lady finger, a major knowledge gap exists regarding the specific phytochemical profiles of the varieties consumed in Ethiopia. Phytochemical concentrations are significantly influenced by genetic cultivars, soil conditions, climate, and postharvest handling [27]. Consequently, global datasets may not accurately reflect the nutritional value of lady fingers available to the Ethiopian population. In Addis Ababa, lady finger is a traditional dietary staple, yet local analytical data remain scarce. Currently, a comparative assessment of produce across the city’s various urban distribution hubs is missing. This study is significant because it accounts for potential variations in nutritional quality resulting from the diverse regional sources and postharvest handling typical of urban market supply chains. Such evidence is essential for formulating accurate dietary recommendations and evaluating this food resource across different urban distribution centers.

This study is positioned as an analytical and comparative market‐based investigation designed to provide a comprehensive assessment of lady finger samples sourced from the major urban hubs of Addis Ababa. The four subcities Arada, Lemi Kura, Bole, and Lafto were selected because they represent the primary geographical and economic distribution centers for lady fingers entering the capital, ensuring a representative survey of what is available to urban consumers. The primary objective of this study was to determine the levels of ascorbic acid (AA), total phenolic content (TPC), total flavonoid content (TFC), and tannin content in lady finger samples from four distinct markets. Furthermore, the study aimed to evaluate their antioxidant activity using three complementary assays (2,2‐diphenyl‐1‐picrylhydrazyl [DPPH], 2,2’‐azino‐bis(3‐ethylbenzothiazoline‐6‐sulfonic acid) [ABTS], and ferric reducing antioxidant power [FRAP]). It was hypothesized that the phytochemical profile and antioxidant potential of lady finger would vary significantly based on the geographical market source, thereby providing a critical baseline for its recognition as a health‐promoting functional food.

## 2. Materials and Methods

### 2.1. Chemicals and Reagents

All the chemicals and reagents utilized in this study were of analytical grade. The standards and reagents used included L‐ascorbic acid (Riedel‐de Haën, Cat: 33034), potassium iodate (KIO_3_, Sigma‐Aldrich, Cat: 215929), potassium iodide (Sigma‐Aldrich, Cat: 32635), and soluble starch (Loba Chemie, Cat: 06126) for iodometric titration. For spectrophotometric phytochemical analyses, gallic acid (Sigma‐Aldrich, Cat: G7384), quercetin (Sigma‐Aldrich, Cat: Q4951), and catechin hydrate (Macklin, Cat: C804968) were used as standards. Reagents included Folin–Ciocalteu’s (Loba Chemie, Cat: 03870), phenol reagent, sodium carbonate (Merck, Cat: 222321), aluminum chloride (Uni‐Chem, Cat: GD11030591), and a vanillin–HCl reagent prepared with 8% HCl (India, Laboratory reagent) in methanol (India, Cat: 106012). For the assessment of antioxidant activity, the stable free radicals DPPH (Sigma‐Aldrich, Cat: D9132) and ABTS (Sigma‐Aldrich, Cat: A1888) were used, along with potassium persulfate (Sigma‐Aldrich, Cat: 216224). Trolox (Sigma‐Aldrich, Cat: 238813) was used as the standard for the ABTS assay, and AA was used as the standard for the DPPH assay. The FRAP assay utilized 2,4,6‐tripyridyl‐s‐triazine (TPTZ) (Sigma‐Aldrich, Cat: T1253), ferric chloride hexahydrate (FeCl_3_.6H_2_O) (Loba Chemie, Cat: 03830), and ferrous sulfate (FeCl_4_.7H_2_O) (Loba Chemie, Cat: 03830) as the standard, with a 300 mmol/L acetate buffer prepared from sodium acetate trihydrate (Sigma‐Aldrich, Cat: S31660) and acetic acid.

### 2.2. Sample Collection and Preparation

Lady finger (*Abelmoschus esculentus*) pods were procured from four distinct and geographically separate open‐air markets within Arada, Lemi Kura, Bole, and Lafto subcities of Addis Ababa, Ethiopia. The samples consisted of fresh, green pods approximately 6–10 cm in length, representing the typical stage for human consumption. To ensure biological replication, 3 kg of fresh lady finger was purchased from three independent vendors (*n* = 3) in each market. The samples from each market were pooled and homogenized to form a composite sample for that specific location. All subsequent analytical measurements were performed in triplicate on these composite samples, representing technical replicates. After collection, the lady fingers were transported to the laboratory for immediate processing. To remove any adhering soils particles and surface contaminants, the samples were first washed thoroughly under running tap water, followed by deionized water. The cleaned pods were then uniformly sliced using a stainless‐steel knife. The prepared lady finger slices were spread onto clean trays and subjected to air‐drying in the shade at ambient room temperature for a period exceeding 2 weeks. This gentle drying method was chosen to minimize the potential thermal degradation of heat‐sensitive phytochemicals, particularly vitamin C, which occurs rapidly above 40°C [23], while vitamin C is sensitive to prolonged air exposure. The average ambient temperature of the environment (January–February) was nearly 20°C–24°C, and the relative humidity was approximately 50%–85%. Once completely dried and became brittle, the lady finger slices were pulverized into fine, homogeneous powder using a ceramic mortar and pestle. To ensure uniform particle size for subsequent extractions, the resulting powder was systematically sieved through a 600‐μm stainless steel standard sieve, followed by a second sieving through FB‐2 sieve with a 500‐μm aperture. The final homogenized powders were stored in sealed, airtight containers in a cool, dark place until analysis.

A precisely weighed 2.5 g portion of each dried lady finger powder was transferred into a flask containing 50 mL of methanol. The flasks were placed on an incubator shaker (Model: ZHWY‐103B, China) and allowed to macerate for a 24‐h period at room temperature. Following maceration, the mixtures were filtered through 110‐mm Whatman filter paper to separate the extract from the solid plant material. The methanol was then evaporated from the filtrate at room temperature in a fume hood to yield the crude extract. These extracts were carefully collected and stored for subsequent phytochemical and antioxidant analysis. For AA determination, an aqueous extract was prepared by weighing exactly 1.0 g of each sieved powder into a 100‐mL volumetric flask and mixing it with 100 mL of deionized water, followed by shaking, centrifugation at 3000 rpm for 15 min, and filtration.

### 2.3. Determination of AA

The AA content was quantified using a direct iodometric titration method with modifications from established procedures [23, 28]. Iodometric titration was selected over chromatographic methods due to its cost‐effectiveness, high precision in routine food analysis, and proven reliability for determining AA in fresh samples within regional laboratory settings [29]. Although chromatographic techniques offer higher sensitivity, iodometric titration remains a widely accepted standard in food composition studies and provides reliable results for comparative assessments.

The iodine solution was prepared using potassium iodate and potassium iodide in an acidic medium. It was standardized against a 3.97 × 10^−4^ M L‐ascorbic acid standard solution (equivalent to 70 mg/L) using a 0.5% starch solution as an indicator. Through this standardization process, the concentration of the iodine solution was determined to be (6.913  ± 0.27) × 10^−3^ M.

For sample analysis, 20 mL of the clear, filtered aqueous lady finger extract was mixed with 1.5 mL of the starch indicator and titrated against the standardized iodine solution. The endpoint was identified by the first appearance of a persistent dark blue‐black color. All titrations were performed in triplicate for each sample to ensure reproducibility.

### 2.4. Determination of TPC

The TPC of the methanolic extracts was determined using the Folin–Ciocalteu colorimetric method [30]. An aliquot of 0.1 mL of the diluted extract was combined with 1 mL of a 10‐fold diluted Folin–Ciocalteu reagent. After 8‐min incubation, 1 mL of a 7.5% sodium carbonate solution was added, and the mixture was allowed to stand for 90 min at room temperature. The absorbance of the resulting blue‐colored complex was measured at 765 nm using a spectrophotometer (PerkinElmer Lambda 950 UV/Vis/NIR). A calibration curve was constructed using gallic acid as the standard (0.02–0.1 mg/mL). The measurements were conducted in triplicate for each sample.

### 2.5. Determination of TFC

The TFC was quantified using the aluminum chloride colorimetric assay [31] with minor modifications. This method is based on the formation of a stable, yellow‐colored complex between flavonoids and aluminum chloride. An equal volume of extract (1 mg/mL) was mixed with an equal volume (2 mL) of 2% aluminum chloride solution. The mixture was then incubated for 1 h at room temperature. The absorbance was measured at 415 nm against a blank sample consisting of the extract and methanol without aluminum chloride. Quercetin was used to generate a standard calibration curve (0.025–0.15 mg/mL). The measurements were conducted in triplicate for each biological replicate.

### 2.6. Determination of Tannin Content

The tannin content was determined using the vanillin–HCl spectrophotometric method, adapted from Zerihun et al. [13]. One gram of the ground sample was extracted with 8 mL of 8% HCl in methanol and centrifuged at 4000 rpm for 10 min. A 1‐mL aliquot of the supernatant was transferred to a test tube, followed by the addition of 5 mL of the vanillin–HCl reagent. After a 20‐min incubation period at room temperature, the absorbance was measured at 500 nm against a blank containing the extract and 8% HCl in methanol without the vanillin reagent.

A standard curve was prepared using catechin (0.2–1.0 mg/mL). Catechin was utilized as the reference standard as it is a well‐characterized monometric unit for quantifying the condensed tannins typically found in the Malvaceae family [25]. While tannins are structurally diverse, catechin provides a practical and widely accepted reference for spectrophotometric quantification. All measurements were conducted in triplicate for each sample.

### 2.7. Antioxidant Activity Assays

Three complementary assays (DPPH, ABTS, and FRAP) were conducted using consistent extract concentrations across all samples. For each assay, three technical replicates were measured for every biological replicate (*n* = 3). For the DPPH, L‐ascorbic acid was chosen as the reference standard to allow for a direct comparison with the primary endogenous antioxidant of the sample. The limit of detection (LOD) and limit of quantification (LOQ) for the DPPH method were determined to be 54 μg/100 g and 164 μg/100 g, respectively. Trolox was utilized as the standard for the ABTS assay to ensure consistency with internationally recognized antioxidant capacity units. The IC_50_ values were calculated for DPPH and ABTS using linear regression analysis by plotting the percentage of inhibition against the extract concentration (*y* = *mx* + *b*).

#### 2.7.1. DPPH Radical Scavenging Assay

The DPPH radical scavenging activity of the lady finger extracts was evaluated according to the method described by Belete et al. [23]. An aliquot of 2 mL of each diluted extract was mixed with 4 mL of a 0.04 mg/mL methanolic solution of DPPH. The reaction mixture was incubated in the dark at room temperature for 30 min. The scavenging of the DPPH radical was determined by measuring the decrease in absorbance at 517 nm. The antioxidant potential was expressed as the 50% inhibitory concentration (IC_50_), representing the concentration of the extract required to scavenge 50% of the DPPH radicals.

#### 2.7.2. ABTS Radical Scavenging Assay

The ABTS radical scavenging activity was determined using a modified procedure from Shah and Modi [32]. The ABTS radical cation (ABTS^·+^) was generated by reacting a 7.4 mM ABTS stock solution with 2.6 mM potassium persulfate and allowing the mixture to stand in the dark for 12–16 h. The ABTS^·+^ solution was then diluted with methanol to achieve a target absorbance at 734 nm. For the assay, 0.4 mL of the extract was mixed with 3 mL of the diluted ABTS^·+^ solution. Absorbance was measured at 734 nm exactly 120 min after the initial mixing.

#### 2.7.3. FRAP Assay

The FRAP of the extracts was assessed following the procedure described by Shah and Modi [32]. This assay measures the ability of an antioxidant to reduce a ferric‐tripyridyltriazine (Fe^3+^‐TPTZ) complex to its ferrous (Fe^2+^) form. The FRAP reagent was freshly prepared by mixing 25 mL of 300 mmol/L acetate buffer (pH = 3.6), 2.5 mL of 10 mmol/L TPTZ solution (in 40 mmol/L HCl), and 2.5 mL of 20 mmol/L aqueous ferric chloride hexahydrate. An aliquot of the extract was mixed with the FRAP reagent and allowed to stand for 30 min in the dark. The absorbance of the resulting blue‐colored product was measured at 593 nm, and a standard curve was created using ferrous sulfate.

### 2.8. Mechanism of Antioxidant Activity

Based on the parameters studied, the antioxidant activity of Ethiopian lady finger is likely to follow a dual pathway mechanism: (i) The chemical mechanism of lady finger phenolics (ArOH) act via hydrogen atom transfer (HAT) to stabilize the DPPH radical: DPPH^·^ + ArOH⟶DPPH‐H + ArO^·^. The resulting phenoxyl radical (ArO·) is stabilized by the resonance of the aromatic ring, effectively stopping the free radical chain reaction. (ii) Single electron transfer (SET): Flavonoids and AA facilitate the reduction of ferric (Fe^3+^) to ferrous (Fe^2+^) ions (Fe^3+^⟶Fe^2+^), as measured in the FRAP assay, by acting as electron donors.

### 2.9. Statistical Analysis

All the analytical determinations were performed in triplicate, and the experimental data were expressed as the mean ± standard deviation (SD). To determine if there were statistically significant differences in the phytochemical content and antioxidant activity among the samples from the four different markets, the data were subjected to analysis of variance (ANOVA), followed by Tukey’s post hoc test to identify significant differences between market sources. A probability value (*p*) of less than 0.05 was considered. All data analysis was performed using Microsoft Excel.

The IC_50_ values, representing the concentration required to inhibit 50% of radicals, were calculated using linear regression analysis by plotting the percentage of inhibition against the extract concentration (*y* = *m* *x* + *c*).

Limit of detection (LOD) = 3.3σ/*S*, limit of quantification (LOQ) = 10*σ*/*S*, where *σ* is the intercept, and *S* is the slope of the curves. The formula for Pearson correlation (*r*) is as follows:

(1) r=∑xi−x¯yi−y¯∑xi−x¯2∑yi−y¯2,

where *x*_*i*_ and *y*_*i*_ are individual data points, and $\overset{¯}{x}$ and $\overset{¯}{y}$ are the average (mean) of data. ∑ means sum up all the values.

### 2.10. Analytical Precision and Recovery of AA

The reliability of the analytical method was supported through triplicate analyses for each sample. The precision of these measurements was evaluated by calculating the SD and relative SD. The relative SD values below 10% are generally considered acceptable as stated by Abajihad and Chandravanshi [28]. In the present study, % RSD values were found to be below 5%, indicating good analytical precision. The relative SDs for the AA measurements in all the four lady finger samples were calculated to be between 2.30% and 4.87%. As these values fall comfortably below the 5% threshold, the method suggests precision and yielded consistent, reproducible results.

### 2.11. Calibration and Method Validation for Determination of Total Phenols, Flavonoids, and Tannins

To ensure the reliability of the phytochemical quantification, the performance characteristics of the analytical methods were evaluated. This involved assessing linearity, sensitivity, and recovery for TPC, TFC, and total tannin content (TTC).

The TPC was determined using a gallic acid standard curve (0.02–0.1 mg/mL), yielding a linear regression equation of *y* = 9.6605*x* + 0.0684 with a high coefficient of determination (*R* ^2^ = 0.9936). For the TFC, a calibration curve was established using quercetin standards (0.025–0.15 mg/mL), resulting in the equation *y* = 6.4946*x* + 0.2098, which indicated excellent linearity (*R* ^2^ = 0.9898). Similarly, the method demonstrated strong linearity across the tested range of tannin concentrations using catechin standards (0.20–1.0 mg/mL), with a correlation coefficient (*R* ^2^ = 0.9934), which demonstrates the method’s suitability for quantitative analysis.

The LOD and LOQ for this method were determined to the TPC, TFC, and TTC, such as the LOD 23.3, 106.6 and 30.1 μg/mL, while the LOQ 70.8, 323 and 91 μg/mL, respectively. These parameters suggest that the colorimetric techniques employed were sufficiently sensitive to detect the phytochemical levels present in the lady finger extracts.

### 2.12. Evaluation of Antioxidant Activity

The antioxidant potential was evaluated using three complementary assays: DPPH, ABTS, and FRAP assay. To ensure accurate quantification, calibration curves were established. The DPPH assay demonstrated strong linearity (*R* ^2 ^ = 0.9976), where a decrease in absorbance corresponded to the reduction of the DPPH radical. Similarly, the ABTS scavenging activity established using Trolox standards showed excellent correlation (*R* ^2 ^ = 0.9981). The performance of the FRAP assay, which measures the capacity to reduce ferric ions to the ferrous form, was verified using a ferrous sulfate standard curve (*R* ^2 ^ = 0.9949). These high correlation coefficients supported that the methods were suitable and consistent for assessing the antioxidant power of lady finger extracts.

## 3. Results and Discussion

### 3.1. AA Content of Lady Finger

#### 3.1.1. Determination of AA Content in Lady Finger

The AA content of the four lady finger (*Abelmoschus esculentus*) samples was evaluated using iodometric titration (Table 1). The results of the analysis showed that the AA content varied significantly among the four samples (*p* < 0.05). The Lemi Kura sample exhibited the highest mean AA concentration (5.90 ± 0.14 mg/100 g), which was significantly higher than the Bole sample, which recorded the lowest concentration (5.27 ± 0.15 mg/100 g).

**TABLE 1:** Ascorbic acid (AA) content of lady finger determined by iodometric titration.

Sample	Concentration of AA (mean ± SD) mM	Mass in mg/100 g (mean ± SD)	% RSD
Lemi Kura	0.3350 ± 0.0076	5.90 ± 0.14^a^	2.30
Bole	0.2966 ± 0.0082	5.27 ± 0.15^b^	2.77
Arada	0.3342 ± 0.016	5.88 ± 0.29^a^	4.87
Lafto	0.3053 ± 0.008	5.38 ± 0.14^b^	2.67

*Note:* Data are presented as mean ± SD of triplicate analysis. Means with different superscript letters within the same column are significantly different (*p* < 0.05).

To assess accuracy, the standard addition technique was employed. The recovery for the Bole sample was found to be 116.8% (Table 2). Although this meets the 80%–120% criteria [29], it suggests a marginal overestimation. This is likely due to the high concentration of mucilage. Chemically, these complex polysaccharides can increase the viscosity of the medium, potentially interfering with the sharp detection of the starch–iodine endpoint. Additionally, other reducing components in the lady finger matrix, such as simple sugars, may cross‐react with iodine, contributing to the higher recovery. Conversely, the Lafto sample recorded the lowest recovery (87.06  ±  0.27%). Such differences likely stem from varying extraction efficiencies and matrix compositions.

**TABLE 2:** Recovery percent of ascorbic acid (AA) for spiked samples.

Sample	Concentration of AA (mean ± SD) mM	Mass in mg/100 g (mean ± SD)	Recovery (%)
Lafto	0.3226 ± 0.013	5.682 ± 0.23	87.06 ± 0.27
Lemi Kura	0.3548 ± 0.0065	6.250 ± 0.12	99.94 ± 0.30
Bole	0.3226 ± 0.013	5.682 ± 0.23	116.8 ± 0.61
Arada	0.3549 ± 0.0065	6.223 ± 0.10	96.8 ± 0.27

*Note:* Data are presented as mean ± SD of triplicate analysis.

#### 3.1.2. Comparative Analysis and Nutritional Significance

The AA levels remained within a relatively narrow range (5.3–5.9 mg/100 g), despite the statistical variations observed among the four subcities. These values are notably lower than the 16–47 mg/100 g typically reported for fresh lady finger in the literature [33–36]. This discrepancy appears to be associated with the extended air‐drying period of 2 weeks. AA is highly sensitive to light, oxygen, and endogenous enzymes; therefore, while shade‐drying avoids high‐temperature degradation, prolonged exposure to atmospheric oxygen may have facilitated the irreversible oxidation of L‐ascorbic acid to dehydroascorbic acid and subsequently into nonbiological diketogulonic acid.

From a nutritional perspective, although these levels are lower than those reported for fresh samples and other fruits such as apples (21.0 mg/100 g) [28], lady finger may still serve as a supplementary source of AA in the Ethiopian diet, particularly when consumed as part of a varied vegetable intake. However, the reduction observed during the drying process highlights the potential importance of minimizing processing and storage time to better preserve micronutrient density.

#### 3.1.3. Methodological Considerations and Limitation

The analytical methods employed in this study provided high precision and reproducible results; however, certain methodological constraints and matrix effects must be considered. While the high mucilage content of lady finger may complicate analytical measurements like iodometric titration, it is also a key bioactive component. As reviewed by Das et al. [8], lady finger polysaccharides and mucilage are being investigated for their mucoadhesive properties in drug delivery and their role in modulating glucose absorption in the gut. This highlights the dual nature of the lady finger matrix, where the same components that interfere with in vitro assays may contribute to the plant’s overall biological relevance and health‐promoting properties. Additionally, the choice of air‐drying as a methodological constraint likely accelerated the oxidative degradation of AA, accounting for the lower values observed compared to fresh samples in literature. Recognizing these matrix‐induced interferences and procedural limits is vital for a balanced interpretation of the phytochemical data, showing that postharvest handling significantly dictates the final nutritional value available to consumers.

### 3.2. Determination of Total Phenolic, Flavonoid, and Tannin Content

Phytochemical contents of lady finger are given in Table 3. Phenolic compounds play a crucial role in antioxidant activities, with significant differences observed among the samples. The Arada sample exhibited the highest phenol content at 130.2 ± 1.4 GAE mg/100 g, while the Bole sample had the smallest phenol content of 98.39 ± 2.1 GAE mg/100 g. High levels of phenolic compounds are associated with various health benefits, including a reduced risk of chronic diseases. The lower levels found in other samples may indicate variations in cultivation practices and genetic differences among the lady finger plants.

**TABLE 3:** Phytochemical content of lady finger samples from four subcities of Addis Ababa.

Sample	Phenol content GAE mg/100 g	Flavonoid content QE mg/100 g	Tannin content catechin mg/100 g
Arada	130.19 ± 1.41^b^	243.92 ± 1.51^b^	62.37 ± 2.21^a^
Lafto	107.08 ± 1.83^a^	211.15 ± 1.42^a^	70.51 ± 2.17^a^
Bole	98.39 ± 2.13^a^	213.65 ± 2.11^a^	69.81 ± 1.49^a^
Lemi Kura	101.69 ± 4.00^a^	171.89 ± 4.98^b^	67.76 ± 0.35^a^

*Note:* Data are presented as mean ± SD of triplicate analysis. Means with different superscript letters within the same column are significantly different (*p*  < 0.05).

**FIGURE 1:**
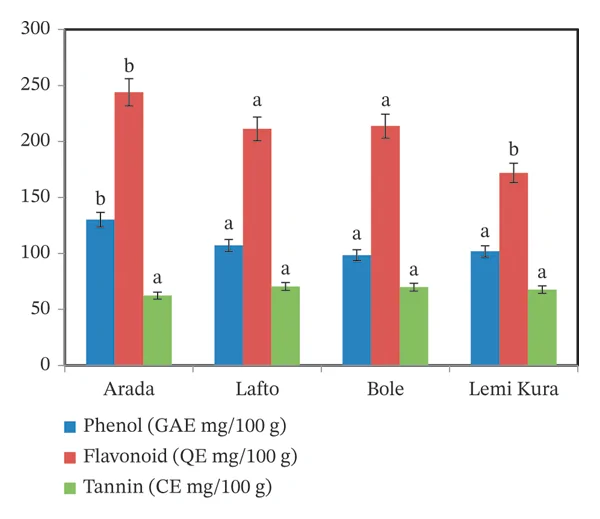
Comparison of total phenolic (TPC), total flavonoid (TFC), and tannin contents of the lady finger samples. Bars with different letters indicate significant differences at *p* < 0.05.

Flavonoids, another important group of phytochemicals, were also most abundant in the Arada sample, which contained 243.9 ± 1.5 QE mg/100 g. This highlights the potential of lady finger as a dietary source of flavonoids, known for their anti‐inflammatory and cardioprotective effects. Variations in flavonoid content across samples reflect the influence of local agricultural practices or climatic conditions. Tannins, while beneficial in moderation, can inhibit the absorption of certain nutrients; their content ranged from 62.37 ± 2.21 mg/100 g in the Arada sample to 70.51 ± 2.17 mg/100 g in the Lafto sample. The higher tannin levels contribute to their antioxidant potential and also carry antinutritional implications. They are known to form insoluble complexes with essential minerals, particularly nonheme iron and calcium, reducing their bioavailability in the digestive tract. Frequent consumption of lady finger varieties with high tannin content could potentially interfere with mineral absorption. However, the traditional Ethiopian cooking practices, such as boiling or stewing with other vegetables, may mitigate the effects by partially leaching these polyphenolic inhibitors. The distinct phytochemical profiles of the major phenolic classes across the four market samples are compared in the bar chart presented in Figure 1.

#### 3.2.1. Comparison of Phytochemicals (Phenolic, Flavonoid, and Tannin Content) of Lady Finger

As shown in Table 4, Açikgöz et al. [30] reported a TPC of 157.8 mg GAE/100 g and Al‐Dabbas et al. [27] reported 167.1 mg GAE/100 g lady fingers. In contrast, the TPC in the lady finger samples from this study ranged from 96.26 to 131.3 mg GAE/100 g, which is higher than the 4.66–49.93 mg GAE/100 g reported by Gemede et al. [35]. Various factors, including extraction techniques, storage conditions, duration, and temperature, can influence the observed variations in phenolic content. While storage conditions can lead to the degradation of phenolic compounds, extraction methods significantly affect their yield, highlighting the need for standardized procedures in phenolic measurement for accurate cross‐study comparisons. In this study, the TFC in lady finger samples ranged from 166.9 to 245.4 mg QE/100 g. The lady finger samples of this investigation had a TTC ranging from 60.16 to 71.30 mg/100 g, which is higher than the 4.93–9.3 mg/100 g reported by Gemede et al. [12], 3.7–6.3 mg/100 g by Okonwu and Ugiomoh [36], and 6.4–6.7 mg/100 g by Andualem [38], but lower than the 332.8 mg/100 g reported by Okanya et al. [37]. These differences appear to be associated with environmental growth conditions and genetic diversity among lady finger varieties, whereas extraction methods, solvents, and extraction duration may affect tannin content. These findings underscore the complexity of measuring tannin content and suggest that future research should employ standardized procedures to ensure comparability of results.

**TABLE 4:** The comparison of phytochemicals in lady finger of the present study with previous reported data.

Phytochemicals	Reported value	References
Phenol (mg GAE/100 g)	157.8	[30]
	4.66–49.93	[35]
	167.1	[27]
	96.26–131.3	This study

Flavonoid (mg QE/100 g)	105.75	[27]
	166.9–245.4	This study

Tannin (mg CE/100 g)	332.8	[37]
	4.93–9.30	[12]
	3.7–6.3	[36]
	6.4–6.7	[38]
	60.16–71.3	This study

#### 3.2.2. Radical Scavenging Potential

**FIGURE 2:**
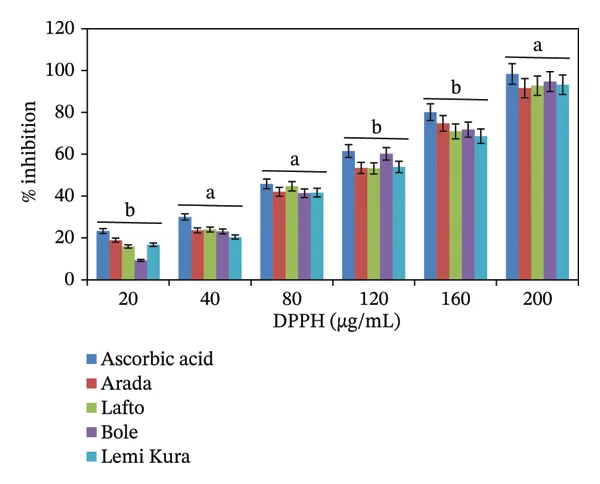
Percentage of inhibition of lady finger with DPPH assay. Bars with different letters indicate significant differences at *p* < 0.01.

All the lady finger samples exhibited robust, dose‐dependent radical scavenging abilities. In the DPPH assay (Figure 2), at a concentration of (200 μg/mL), inhibition reached up to 94.73%. The sample from Bole showed the highest scavenging activity (94.73 ± 0.0986%), followed by Lemi Kura (93.23 ± 0.036%), while Lafto and Arada samples had slightly lower activity at (92.77 ± 0.04%) and (91.59 ± 0.5374%), respectively. These results may suggest that variations in specific phenolic or flavonoid compositions across subcities influence the effectiveness of free radical neutralization.

**FIGURE 3:**
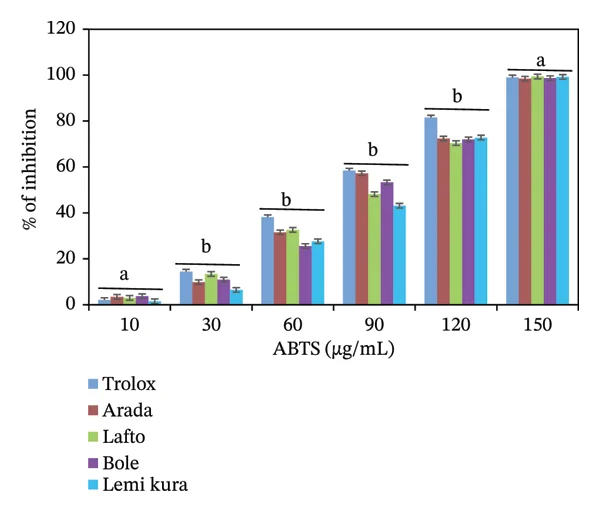
Percentage of inhibition of lady finger with ABTS assay. Bars with different letters indicate significant differences at *p* < 0.001.

The ABTS assay (Figure 3) further supported these findings, with radical inhibition values exceeding at the highest concentration of (150 μg/mL). The Arada sample exhibited a clear dose‐dependent increase in scavenging activity, reaching a maximum inhibition (98.4488 ± 0.0761%). Similarly, the Lafto sample demonstrated high potency, achieving at the same concentration. This steady increase in inhibition (99.39 ± 0.0457%), which closely comparable to the Trolox standard, suggests a high antioxidant potential. Furthermore, the Bole sample demonstrated significant scavenging efficacy, with inhibition values (98.70 ± 0.0171%) and Lemi Kura with the inhibition value (99.2348 ± 0.0086%). This appears associated with the presence of potent antioxidants capable of mitigating oxidative stress. The radical scavenging activities at maximum concentrations were nearly identical across all subcities, indicating that lady finger samples in Addis Ababa are a consistent and beneficial source of natural antioxidants.

#### 3.2.3. FRAP

**FIGURE 4:**
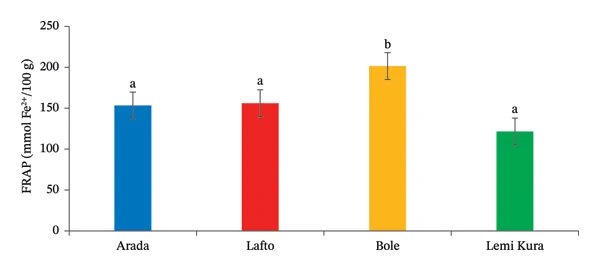
The reducing power of lady finger samples. Different letters denote statistical significance (*p* < 0.05).

The FRAP assay (Figure 4) was used to assess the electron‐donating capacity of the extracts. The results suggest significant antioxidant potential, with the Bole sample exhibiting the highest antioxidant activity (201.4 ± 7.1 mmol Fe^2+^/100 g ), while the Lemi Kura sample appeared to have the lowest (121.5 ± 1.2 mmol Fe^2+^/100 g). The FRAP values reflect the ability of the lady finger samples to donate electrons. In biological systems, the reducing power is the fundamental mechanism against oxidative stress, as it can reduce pro‐oxidant metal ions to less reactive forms, thereby preventing the Fenton reaction which generates highly damaging hydroxyl radicals.

#### 3.2.4. Values and Comparative Potency

All the four lady finger extracts exhibited strong radical scavenging activity in both the DPPH and ABTS assays. In the DPPH assay, the scavenging capacity, reflected by the IC_50_ value (the concentration needed to inhibit 50% of radicals), ranged from 84.4 to 86.7 μg/mL, with the Bole sample showing the highest activity at an IC_50_ value of 84.4 ± 0.2 μg/mL. The ABTS assay also demonstrated robust dose‐dependent scavenging, with IC_50_ values ranging from 63.9 to 92.5 μg/mL. The Arada and Bole samples were the most effective, achieving the lowest IC_50_ values of 63.9 and 64.8 μg/mL, respectively, closely matching the Trolox standard (IC_50_ = 63.2 μg/mL), indicating high antioxidant efficacy. The in vitro antioxidant activities of lady finger samples are given in Table 5.

**TABLE 5:** In Vitro antioxidant activity of lady finger samples.

Sample	DPPH IC_50_	ABTS IC_50_
Arada	86.7 ± 0.2^a^	63.9 ± 0.1^a^
Lafto	85.9 ± 0.5^a^	90.9 ± 0.9^b^
Bole	84.4 ± 0.2^b^	64.8 ± 0.2^a^
Lemi Kura	86.5 ± 0.3^a^	92.5 ± 0.1^c^

*Note:* Data are presented as Mean ± SD (μg/mL). Means with different superscript letters within the same column are significantly different (*p* < 0.05).

#### 3.2.5. Comparison of Antioxidant Capacity of Lady Finger by DPPH, ABTS, and FRAP Assay

The comparison of antioxidant activity in lady finger of this study with previously reported data is presented in Table 6. The DPPH assay yielded IC_50_ values ranging from 84.4 to 86.7 μg/mL, which are significantly lower than earlier findings by Celebi et al. [14], who reported an IC_50_ of 431.45 μg/mL, and Al‐Dabbas et al. [27], who noted a range of 3810–134,000 μg/mL. Additionally, Gemede et al. [12] reported IC_50_ values between 3150 and 6600 μg/mL. These variations in antioxidant activity highlight the influence of factors such as extract concentration, storage duration, and temperature on the phytochemical composition and antioxidant potential of lady finger. The ABTS test results indicated IC_50_ values from 63.2 to 92.5 μg/mL, which are higher than those reported by Faisal and Handayani [40] (24.5–49.54 μg/mL) and comparable to Gong et al. [39], who found values ranging from 50 to 2870 μg/mL. The IC_50_ values obtained indicate significantly higher antioxidant potency compared to several studies where IC_50_ values exceeded 1000 μg/mL. This enhanced sensitivity is likely associated with the use of methanol as an extraction solvent, which efficiently solubilizes polar phenolic compounds and flavonoids compared to aqueous solvents. Furthermore, variations in these results may stem from cultivar specific characteristics of Ethiopian lady finger, environmental growth conditions in Addis Ababa, and differences in postharvest handling.

**TABLE 6:** Comparison of antioxidant activities of lady finger of the present study with previously reported data.

Method of assay	Antioxidant (μg/mL)	Reference
DPPH (IC_50_)	431.5	[14]
	3810–134,000	[27]
	3150–6600	[12]
	84.4–86.7	This study

ABTS (IC_50_)	50–2870	[39]
	24.5–49.54	[40]
	63.9–92.5	This study

*mmol* *Fe* ^2+^ */100 g of extract*		
FRAP assay	28.47–92.72	[39]
	121.5–201.4	This study

*Note:* IC_50_ values for DPPH and ABTS are expressed in μg/mL; FRAP values are expressed in mmol Fe^2+^/100 g of extract.

The observed differences between the DPPH and ABTS results may be attributed to their distinct chemical mechanisms. The DPPH radical is a sterically hindered, nitrogen‐centered radical that primarily reacts via the HAT mechanism. In contrast, the ABTS assay is more versatile, operating through both HAT and SET pathways. Furthermore, the ABTS cation radical is amphiphilic soluble in both aqueous and organic phases, allowing it to react with a broader spectrum of hydrophilic and lipophilic antioxidants within the lady finger matrix. This appears to explain why the ABTS IC_50_ values were generally lower than the DPPH values.

Regarding the FRAP assay, unlike the radical scavenging methods, it measures reducing power exclusively via the SET mechanism. The high reduction capacity recorded suggests that lady finger phenolics are effective electron donors. In biological systems, this mechanism is crucial as it can reduce pro‐oxidant metal ions to less reactive forms, potentially hindering the formation of highly damaging hydroxyl radicals via the Fenton reaction.

#### 3.2.6. Correlation Analysis

Pearson correlation analysis (*n* = 12, based on 4 samples × 3 replicates) provides insight into the phytochemicals responsible for these activities. A strong positive correlation was found between TPC and TFC (*r* = 0.75, *p* < 0.05), indicating that these compounds are the major constituents of the lady finger extract.

The strong negative correlation between TFC and ABTS IC_50_ (*r* = −0.759, *p* < 0.01) suggests that flavonoids may be the fundamental contributor to the radical scavenging capacity. The structural configuration of flavonoids, specifically the presence of hydroxyl groups on the B‐ring, likely facilitates the stabilization of radicals through electron resonance. Conversely, a moderate positive correlation between TPC and DPPH IC_50_ (*r* = 0.636) indicates that while total phenolics contribute to scavenging, they may operate through a slower HAT mechanism compared to specific flavonoids. This suggests that while the total pool of phenolics contributes to overall antioxidant capacity, the specific structural motifs of flavonoids are more efficient at stabilizing radical species, explaining their dominant role in the ABTS results. The weak correlation between tannin content and FRAP (*r* = 0.301) suggests that tannins, while present, may not be the primary electron‐donating species under the acidic conditions(pH = 3.6) of the FRAP assay.

While this study provides a vital baseline for the nutritional mapping of Ethiopian lady finger, its geographical scope was limited to four subcities in Addis Ababa. This restriction limits the broader applicability of the findings, as diverse agro‐ecological factors across Ethiopia such as soil composition, altitude, and rainfall significantly influence phytochemical biosynthesis. Furthermore, the absence of in vivo validation means that the observed antioxidant potential has not been confirmed within complex biological systems, leaving the actual physiological impact of the crop uncertain.

Additionally, the reliance on in vitro assays and colorimetric methods fails to account for gastrointestinal bioavailability or the specific molecules responsible for the observed activity. Without advanced characterization, such as HPLC–MS to isolate individual phenolic acids or flavonoids, the biological relevance of these broad phytochemical groups remains speculative. Future research should prioritize high‐resolution profiling and clinical dose–response studies to determine how metabolic transformations affect these compounds and confirm whether laboratory results translate into tangible health outcomes.

## 4. Conclusion

This study achieved its primary objective of providing a comparative analytical assessment of lady finger samples sourced from various markets in Addis Ababa, contributing a baseline for the nutritional mapping of Ethiopian vegetables. The findings suggest that the market source may influence the nutritional quality of the produce, as evidenced by significant variations in phytochemical levels across the four subcities. Specifically, while the Arada sample exhibited the highest total phenolic and flavonoid content and the Lemi Kura sample was found to be the richest source of AA, the Bole sample demonstrated the strongest overall antioxidant activity across the assays. This is likely due to its unique profile and potential synergistic effects of its phenolic and tannin constituents.

The study further supports the reliability and suitability of accessible colorimetric and titrimetric methods for routine food quality monitoring in a resource limited setting. Based on the high in vitro antioxidant potential, lady finger appears to show promise as a functional food that may contribute to the mitigation of oxidative stress‐related conditions. Future research should prioritize bioavailability assessments and in vivo validation to support the gastrointestinal stability of these phytochemicals and determine their actual clinical relevance for human health.

## Author Contributions

Gezehagn Tola, Weldegebriel Yohannes, and Bhagwan Singh Chandravanshi designed the study. Gezehagn Tola and Weldegebriel Yohannes were responsible for experimental data collection. Gezehagn Tola was responsible for sample preparation, data analysis, and original draft preparation. Gezehagn Tola, Weldegebriel Yohannes, and Bhagwan Singh Chandravanshi interpreted the data. Bhagwan Singh Chandravanshi edited the manuscript.

## Funding

This research did not receive any specific grant from funding agencies in the public, commercial, or not‐for‐profit sectors.

## Disclosure

All authors read and approved the final manuscript and agreed for its publication.

## Conflicts of Interest

The authors declare no conflicts of interest.

## Data Availability

All the data are included in the manuscript. There are no additional data with the editor.

## References

[1] Zhai M. , Huang G. , Liu L. , Zheng B. , and Guan Y. , Network Analysis of Different Types of Food Flows: Establishing the Interaction Between Food Flows and Economic Flows, Resources, Conservation and Recycling. (2019) 143, 143–153, 10.1016/j.resconrec.2018.12.016.

[2] Buturi C. V. , Mauro R. P. , Fogliano V. , Leonardi C. , and Giuffrida F. , Mineral Biofortification of Vegetables as a Tool to Improve Human Diet, Foods. (2021) 10, no. 2, 10.3390/foods10020223. PMC7911230 33494459

[3] Hagos M. , Chandravanshi B. S. , Redi-Abshiro M. , and Yaya E. E. , Determination of Total Phenolic, Total Flavonoid, Ascorbic Acid Contents and Antioxidant Activity of Pumpkin Flesh, Peel and Seeds From Different Regions of Ethiopia, Bulletin of the Chemical Society of Ethiopia. (2023) 37, no. 5, 1093–1108, 10.4314/bcse.v37i5.3.

[4] Caluête M. E. E. , de Souza L. M. P. , Ferreira E. D. S. et al., Nutritional, Anti-Nutritional and Phytochemical Status of Okra Leaves (*Abelmoschus esculentus*) Subjected to Different Processes, African Journal of Biotechnology. (2015) 14, no. 8, 683–687, 10.5897/ajb2014.14356.

[5] Haile K. , Mehari B. , Atlabachew M. , and Chandravanshi B. S. , Phenolic Composition and Antioxidant Activities of Cladodes of the Two Varieties of Cactus Pear (*Opuntia ficus-indica*) Grown in Ethiopia, Bulletin of the Chemical Society of Ethiopia. (2016) 30, no. 3, 347–356, 10.4314/bcse.v30i3.3.

[6] Seifu T. , Mehari B. , Atlabachew M. , and Chandravanshi B. S. , Polyphenolic Content and Antioxidant Activity of Leaves of *Urtica simensis* Grown in Ethiopia, Latin American Applied Research. (2017) 47, no. 1, 35–40, 10.52292/j.laar.2017.295.

[7] Ramya V. and Patel P. , Health Benefits of Vegetables, International Journal of Chemical Studies. (2019) 7, no. 2, 82–85.

[8] Das S. , Nandi G. , and Ghosh L. K. , Okra and Its Various Applications in Drug Delivery, Food Technology, Health Care, and Pharmacological Aspects a Review, Journal of Pharmaceutical Sciences and Research. (2019) 11, no. 6, 2139–2147.

[9] Kurutas E. B. , The Importance of Antioxidants Which Play the Role in Cellular Response Against Oxidative/Nitrosative Stress: Current State, Nutrition Journal. (2015) 15, 10.1186/s12937-016-0186-5. PMC4960740 27456681

[10] Chaudhary P. , Janmeda P. , Docea A. O. et al., Oxidative Stress, Free Radicals and Antioxidants: Potential Crosstalk in the Pathophysiology of Human Diseases, Frontiers in Chemistry. (2023) 11, 10.3389/fchem.2023.1158198. PMC10206224 37234200

[11] Xie X. , He Z. , Chen N. , Tang Z. , Wang Q. , and Cai Y. , The Roles of Environmental Factors in Regulation of Oxidative Stress in Plants, BioMed Research International. (2019) 2019, 1–11, 10.1155/2019/9732325.

[12] Gemede H. F. , Haki G. D. , Beyene F. , Woldegiorgis A. Z. , and Rakshit S. K. , Proximate, Mineral, and Antinutrient Compositions of Indigenous Okra (*Abelmoschus esculentus*) Pod Accessions: Implications for Mineral Bioavailability, Food Science and Nutrition. (2015) 4, no. 2, 223–233, 10.1002/fsn3.282. 27004112 PMC4779480

[13] Zerihun M. , Berhe H. , Mulu M. , Argahgn Z. , and Alemu M. D. , Nutritive Composition and Physicochemical Properties and Oil Contents of Okra (*Abelmoschus esculentus* L.) Seed in Middle Awash, Ethiopia, Journal of Food Processing & Technology. (2020) 11, 10.35248/2157-7110.20.11.848.

[14] Celebi B. , Ozer S. , and Ates S. C. , Characterization of Local Okra Seed (*Abelmoschus esculentus*) Extract and Evaluation of Bioactivity, Cumhuriyet Science Journal. (2024) 45, no. 4, 707–712, 10.17776/csj.1519528.

[15] Haffez M. , Hassan S. M. , Mughal S. S. , Munir M. , and Khan M. K. , Antioxidant, Antimicrobial and Cytotoxic Potential of *Abelmoschus esculentus* , Chemical and Biomolecular Engineering. (2020) 5, no. 4, 69–79, 10.11648/j.cbe.20200504.11.

[16] Marcus A. C. and Anyadiegwu V. C. , Studies on the Mineral Contents of the Pod of *Abelmoschus esculentus* Harvested From Some Parts of Rivers State, Nigeria, International Journal of Advanced Research in Computer Science. (2021) 8, no. 1, 1–10, 10.20431/2349-0403.0801001.

[17] Wu D.-T. , Nie X.-R. , Shen D.-D. et al., Phenolic Compounds, Antioxidant Activities, and Inhibitory Effects on Digestive Enzymes of Different Cultivars of Okra (*Abelmoschus esculentus*), Molecules. (2020) 25, no. 6, 10.3390/molecules25061276. PMC7143948 32168896

[18] Graham J. O. , Agbenorhevi J. K. , and Kpodo F. M. , Total Phenol Content and Antioxidant Activity of Okra Seeds From Different Genotypes, American Journal of Food and Nutrition. (2017) 5, no. 3, 90–94, 10.12691/ajfn-5-3-2.

[19] Lopez-Corona A. V. , Valencia-Espinosa I. , González-Sánchez F. A. , Sánchez-López A. L. , Garcia-Amezquita L. E. , and Garcia-Varela R. , Antioxidant, Anti-Inflammatory and Cytotoxic Activity of Phenolic Compound Family Extracted From Raspberries (*Rubus idaeus*): A General Review, Antioxidants. (2022) 11, no. 6, 10.3390/antiox11061192. PMC9230908 35740089

[20] Xie J. , Xiong S. , Li Y. et al., Phenolic Acids From Medicinal and Edible Homologous Plants: A Potential Anti-Inflammatory Agent for Inflammatory Diseases, Frontiers in Immunology. (2024) 15, 10.3389/fimmu.2024.1345002. PMC11224438 38975345

[21] Zheng X. , Zhang X. , and Zeng F. , Biological Functions and Health Benefits of Flavonoids in Fruits and Vegetables: A Contemporary Review, Foods. (2025) 14, no. 2, 10.3390/foods14020155. PMC11765039 39856822

[22] Barreca M. M. , Alessandro R. , and Corrado C. , Effects of Flavonoids on Cancer, Cardiovascular and Neurodegenerative Diseases: Role of NF-ĸB Signaling Pathway, International Journal of Molecular Sciences. (2023) 24, no. 11, 10.3390/ijms24119236. PMC10252664 37298188

[23] Belete A. , Yisak H. , Chandravanshi B. S. , and Yaya E. E. , Ascorbic Acid Content and the Antioxidant Activity of Common Fruits Commercially Available in Addis Ababa, Ethiopia, Bulletin of the Chemical Society of Ethiopia. (2023) 37, no. 2, 277–288, 10.4314/bcse.v37i2.3.

[24] Kasshun F. and Chandravanshi B. S. , Polyphenol Contents of Ten Most Widely Cultivated and Consumed Vegetables in Ethiopia, Bulletin of the Chemical Society of Ethiopia. (2025) 39, no. 6, 1029–1041, 10.4314/bcse.v39i6.1.

[25] Cosme F. , Aires A. , Pinto T. , Oliveira I. , Vilela A. , and Gonçalves B. , A Comprehensive Review of Bioactive Tannins in Foods and Beverages: Functional Properties, Health Benefits, and Sensory Qualities, Molecules. (2025) 30, no. 4, 10.3390/molecules30040800. PMC11858154 40005115

[26] Absakine S. I. , Nutritional and Medicinal Values of the Three Most Used Plants in Chad: *Abelmoschus esculentus*, *Hibiscus sabdariffa*, and *Corchorus olitorius* , American Journal of Plant Sciences. (2024) 15, 636–650, 10.4236/ajps.2024.158043.

[27] Al-Dabbas M. M. , Moumneh M. , Hamad H. J. et al., Impact of Processing and Preservation Methods and Storage on Total Phenolics, Flavonoids, and Antioxidant Activities of Okra (*Abelmoschus esculentus* L.), Foods. (2023) 12, no. 19, 10.3390/foods12193711. PMC10573059 37835364

[28] Abajihad A. and Chandravanshi B. S. , Vitamin C Content and Antioxidant Activity of Eight Selected Vegetables Widely Consumed in Addis Ababa, Ethiopia, Bulletin of the Chemical Society of Ethiopia. (2025) 39, no. 4, 629–641, 10.4314/bcse.v39i4.3.

[29] Abe-Matsumoto L. T. , Sampaio G. R. , and Bastos D. H. M. , Is Titration as Accurate as HPLC for Determination of Vitamin C in Supplements? Titration Versus HPLC for Vitamin C Analysis, American Journal of Analytical Chemistry. (2020) 11, no. 7, 269–279, 10.4236/ajac.2020.117021.

[30] Açikgöz Ç. , Borazan A. A. , Andoğlu E. M. , and Gökdai D. , Chemical Composition of Turkish Okra Seeds (*Hibiscus esculenta* L.) and the Total Phenolic Content of Okra Seeds Flour, Anadolu University Journal of Science and Technology: A Applied Science and Engineering. (2016) 17, no. 5, 766–774, 10.18038/aubtda.279845.

[31] Kim G. N. , Shin M.-R. , Shin S. H. et al., Study of Antiobesity Effect Through Inhibition of Pancreatic Lipase Activity of *Diospyros kaki* Fruit and *Citrus unshiu* Peel, BioMed Research International. (2016) 2016, 2–4, 10.1155/2016/1723042. PMC4978832 27529064

[32] Shah P. and Modi H. A. , Comparative Study of DPPH, ABTS and FRAP Assays for Determination of Antioxidant Activity, International Journal for Research in Applied Science and Engineering Technology. (2015) 3, no. 6, 636–641.

[33] Liu Y. , Qi J. , Luo J. et al., Okra in Food Field: Nutritional Value, Health Benefits and Effects of Processing Methods on Quality, Food Reviews International. (2019) 37, no. 1, 67–81, 10.1080/87559129.2019.1695833.

[34] Sindhu R. K. and Puri V. , Phytochemical, Nutritional and Pharmacological Evidences for *Abelmoschus esculentus* (L.), Journal of Phytopharmacology. (2016) 5, no. 6, 238–241, 10.31254/phyto.2016.5606.

[35] Gemede H. F. , Haki G. D. , Beyene F. , Rakshit S. K. , and Woldegiorgis A. Z. , Indigenous Ethiopian Okra (*Abelmoschus esculentus*) Mucilage: A Novel Ingredient With Functional and Antioxidant Properties, Food Science and Nutrition. (2018) 6, no. 3, 563–571, 10.1002/fsn3.596. 29876107 PMC5980337

[36] Okonwu K. and Ugiomoh I. G. , Tannin Contents of Some Economic Plants in Nigeria, Journal of Plant Sciences. (2015) 10, no. 4, 159–166, 10.3923/jps.2015.159.166.

[37] Okanya A. J. , Audu S. S. , and Igwe O. E. , Evaluation of the Nutritional Quality and Phytochemical Properties of Okra (*Abelmoschus esculentus*, L.) Seed Flour, Nigerian Research Journal of Chemical Sciences. (2021) 9, no. 2, 296–311.

[38] Andualem M. , Nutritional and Anti-Nutritional Characteristics of Okra (*Abelmoschus esculentus* L*.* Moench) Accessions Grown in Pawe District, Northwestern Ethiopia, International Journal of Agriculture and Biosciences. (2023) 12, no. 1, 18–21, 10.47278/journal.ijab/2023.040.

[39] Gong X. , Huang X. , Yang T. , Wen J. , Zhou W. , and Li J. , Effect of Drying Methods on Physicochemical Properties and Antioxidant Activities of Okra Pods, Journal of Food Processing and Preservation. (2019) 43, no. 11, 10.1111/jfpp.14277.

[40] Faisal H. and Handayani S. , Comparison of Antioxidant Activity of Ethanol Extract of Fruit and Okra Leaves (*Abelmoschus esculentus* L. Moench) by DPPH and ABTS Methods, Indonesian Journal of Pharmaceutical and Clinical Research. (2019) 2, no. 2, 6–12, 10.32734/IDJPCR.V2I2.2815.

